# Giant Intrapulmonary Solitary Fibrous Tumour With a Possible Liver Metastasis: A Rare Case

**DOI:** 10.1002/rcr2.70754

**Published:** 2026-10-01

**Authors:** Desdiani Desdiani, Nurjati Chairani Siregar, Asysyukriati Prawiro, Khansa Kiasati Chandra Syahbunan

**Affiliations:** ^1^ Faculty of Medicine and Nutrition IPB University Bogor Indonesia; ^2^ Department of Anatomical Pathology, Faculty of Medicine Universitas Indonesia‐Cipto Mangunkusumo Hospital Jakarta Indonesia; ^3^ Faculty of Medicine UPN Veteran Jakarta Jakarta Indonesia; ^4^ Faculty of Medicine Universitas Airlangga Surabaya Indonesia

**Keywords:** hypoglycaemia, intrapulmonary solitary fibrous tumour, neoplasm metastasis, pericardial effusion, pleural effusion

## Abstract

Intrapulmonary solitary fibrous tumours (SFTs) are exceptionally rare mesenchymal neoplasms, particularly when presenting as giant thoracic masses. We report a case of a 57‐year‐old woman with progressive chest pain and dyspnoea who initially presented with pleural effusion and hypoglycaemia and received empirical anti‐tuberculosis therapy without improvement. Contrast‐enhanced CT revealed a left lower lobe mass enlarging to 11 × 16.5 × 15.2 cm on repeat imaging, with rightward cardiac displacement, pericardial effusion and a hepatic nodule suspicious for metastasis to the liver. Core needle biopsy revealed a low‐grade spindle cell neoplasm. Immunohistochemistry revealed diffuse STAT6 positivity, partial CD34 positivity, Ki‐67 < 5%, and negativity for CK7, desmin, SMA and S100, supporting the diagnosis of SFT. Following a multidisciplinary evaluation, six cycles of systemic chemotherapy were administered, and the patient remained clinically stable. This case highlights the diagnostic challenges of giant intrapulmonary SFT and the pivotal role of STAT6 immunohistochemistry in establishing the diagnosis.

## Graphical abstract

A 57‐year‐old woman presented with a giant intrapulmonary solitary fibrous tumour (SFT) that progressed from a large left lower lobe mass to extensive thoracic disease with cardiac displacement, pericardial effusion and a hepatic lesion suspicious for metastasis. Histopathological and immunohistochemical findings, including diffuse STAT6 positivity, confirmed the diagnosis. Despite the unresectable disease, the patient remained clinically stable following systemic chemotherapy.

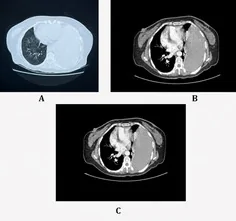

## 1 Introduction

A solitary fibrous tumour (SFT) is a rare mesenchymal neoplasm that most commonly arises from the pleura but may occasionally occur within the lung parenchyma [1]. Intrapulmonary SFT is exceptionally uncommon and typically affects adults aged 50–70 years [2]. Although most tumours follow a benign course, large lesions may cause compressive symptoms and exhibit malignant behaviour [1, 3]. Diagnosis relies on histopathological and immunohistochemical evaluations, with STAT6 and CD34 serving as key diagnostic markers [4]. Complete surgical resection is the preferred treatment, whereas systemic therapy is considered for unresectable or metastatic disease [5]. We report a rare case of a giant intrapulmonary SFT presenting with pleural and pericardial effusions and a hepatic lesion suspicious for metastasis.

## 2 Case Report

A 57‐year‐old woman presented with progressive left‐sided chest pain and shortness of breath. Seven months earlier, a left pleural effusion had been incidentally detected during hospitalization for hypoglycaemia. She had a history of diabetes mellitus, passive smoking exposure and over 20 years of employment in a textile factory. Initial chest radiography revealed a homogeneous opacity in the left lower lung field with pleural effusion. Empirical anti‐tuberculosis treatment was administered for 2 months, but no improvement was observed.

Contrast‐enhanced chest CT performed in July 2025 revealed a large left lower lobe mass measuring 14.1 × 15.6 × 12.1 cm. After being lost to follow‐up for over 5 months, the patient returned with worsening symptoms. Repeat CT demonstrated interval enlargement of the mass to 11 × 16.5 × 15.2 cm, causing rightward cardiac displacement and associated with atelectasis, pericardial effusion, minimal pleural effusion and a 3.8‐cm hepatic nodule suspicious for metastasis (T4N0M1b) (Figure 1A–C). Core needle biopsy revealed a low‐grade spindle cell neoplasm with 1–2 mitoses per 10 high‐power fields. Immunohistochemistry revealed diffuse STAT6 positivity, partial CD34 positivity, Ki‐67 < 5%, and negativity for CK7, desmin, SMA and S100, confirming the diagnosis of a SFT (Figure 2A–F). Following a multidisciplinary evaluation, the patient received six cycles of doxorubicin, cisplatin, vincristine and cyclophosphamide. Follow‐up CT revealed a persistent mass measuring 16.2 × 9.0 × 14.7 cm with suspected pericardial invasion but no mediastinal lymphadenopathy. Pulmonary function testing revealed mild diffusion impairment (DLCO 67%) with preserved spirometry. The patient remained clinically stable following treatment.

**FIGURE 1:**
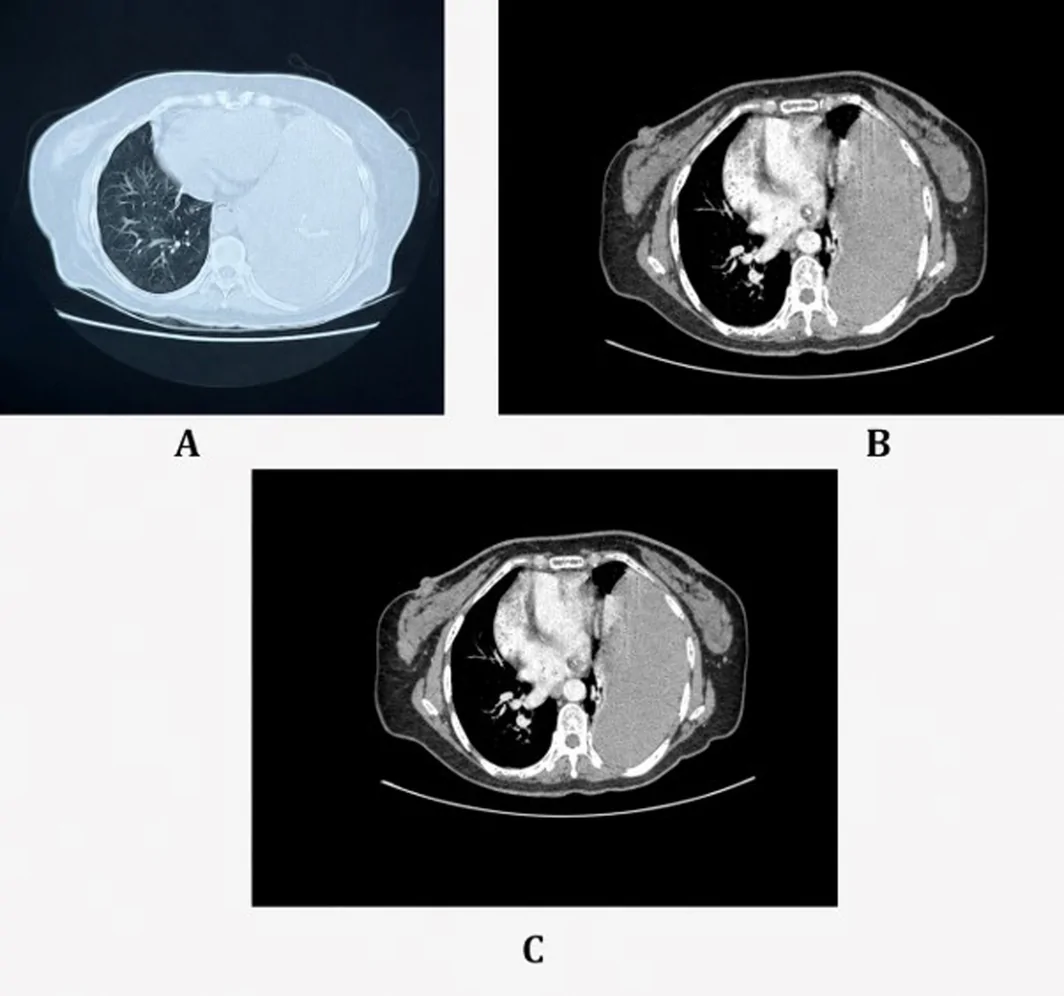
Serial contrast‐enhanced thoracic CT scans illustrating the clinical course of a giant intrapulmonary solitary fibrous tumour. (A) Initial presentation (June 2025) showing a large left lower lobe mass. (B) Disease progression at follow‐up (December 2025) with tumour enlargement, cardiac displacement, pericardial effusion and a hepatic lesion suspicious for metastasis. (C) Post‐chemotherapy assessment (May 2026) demonstrating a persistent tumour burden with suspected pericardial extension and minimal residual pleural effusion.

**FIGURE 2:**
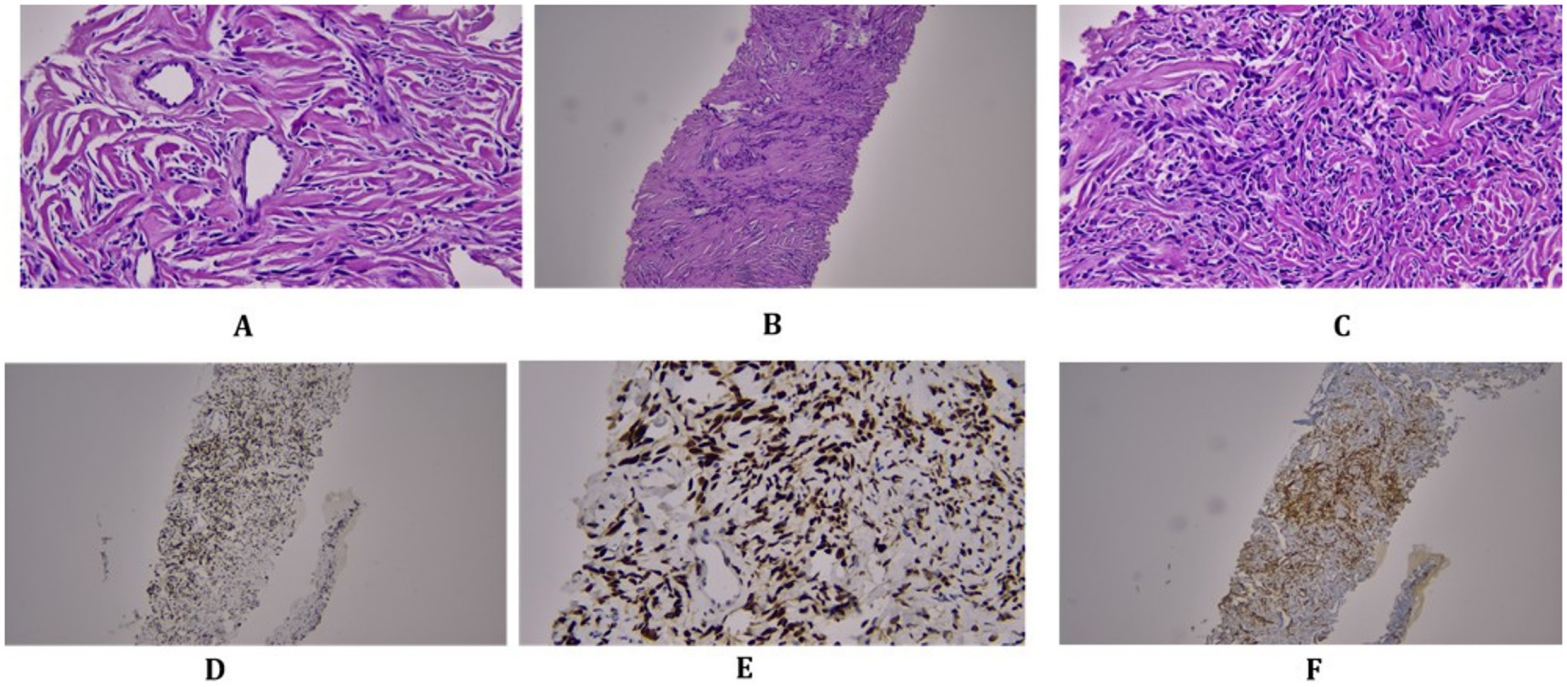
Histopathological and immunohistochemical findings. (A–C) Haematoxylin and eosin‐stained sections demonstrating a spindle cell neoplasm with a patternless architecture, collagenous stroma and hemangiopericytoma‐like vessels. (D, E) Diffuse nuclear STAT6 positivity in the tumour cells. (F) Partial CD34 positivity. These findings are consistent with those of solitary fibrous tumours.

## 3 Discussion

Intrapulmonary SFTs are exceptionally rare mesenchymal neoplasms that most commonly affect individuals in the fifth to seventh decades of life. The clinical manifestations are often nonspecific, including chest pain, dyspnoea, cough or incidental radiographic findings. Consistent with Wang et al., who analysed 52 cases with a mean age of onset of 52.7 years, our patient presented with chest pain and dyspnoea, with the tumour arising in the left lower lobe, the most frequently affected pulmonary location in that series [2]. The concurrent detection of pleural effusion and hypoglycaemia further complicates the diagnostic workup. Hypoglycaemia in the setting of SFT may suggest Doege–Potter syndrome, a rare paraneoplastic condition caused by tumour secretion of insulin‐like growth factor‐2 (IGF‐2), with fewer than 2000 cases reported worldwide [4]. Serum IGF‐2 levels were not assessed in our patient; therefore, formal confirmation of this syndrome was not possible.

Radiologically, pulmonary SFT typically presents as a well‐circumscribed soft‐tissue mass, with the majority of reported tumours measuring less than 10 cm [1]. The tumour in our patient was notably larger, reaching up to 16.5 cm, with associated atelectasis, pericardial effusion, rightward cardiac displacement and a hepatic lesion suspicious for metastasis (T4N0M1b). Aziz et al. reported a comparably large presentation, in which a mass measuring 14 × 12 × 23 cm caused compressive atelectasis and raised concern for hepatic and splenic metastases [1]. The combination of a massive tumour burden, compressive cardio mediastinal effects, suspected pericardial involvement and possible distant spread in our patient represents an unusual and particularly challenging presentation.

Definitive diagnosis relies on histopathological and immunohistochemical evaluations [2]. Core needle biopsy revealed a low‐grade spindle cell neoplasm with 1–2 mitoses per 10 high‐power fields. Immunohistochemistry revealed diffuse STAT6 positivity, partial CD34 positivity and a Ki‐67 index of less than 5%, with negative staining for CK7, desmin, SMA and S100. These findings are consistent with those of Wang et al., who demonstrated positive expression of CD34, STAT6, vimentin, BCL2 and CD99, with a Ki‐67 index of ≤ 5% in borderline tumours [2]. Nuclear STAT6 expression is a highly sensitive and specific marker, particularly useful for distinguishing SFT from other spindle cell neoplasms [2]. Histopathological features associated with malignant behaviour include tumour size greater than 10 cm, necrosis, increased mitotic activity, hypercellularity and nuclear pleomorphism [2, 5]. Although the low mitotic count and Ki‐67 index suggested low‐grade histology, the large tumour size and suspected metastatic disease raised concerns regarding aggressive biological behaviour.

Complete surgical resection remains the treatment of choice for localized SFT. However, given the extent of the disease and suspected metastatic spread, upfront surgical resection was deferred following a multidisciplinary discussion. Although evidence for chemotherapy in advanced SFT remains limited, anthracycline‐based regimens are commonly employed in unresectable or metastatic cases, and targeted therapies, including temozolomide–bevacizumab, pazopanib and sunitinib, have shown promising results in refractory disease [5]. After six cycles of doxorubicin, cisplatin, vincristine and cyclophosphamide, the patient remained clinically stable despite persistent radiological findings. This case highlights the importance of integrating clinical, radiological, histopathological and immunohistochemical findings to establish an accurate diagnosis and guide the individualized management of giant intrapulmonary SFT.

## Author Contributions

**Desdiani Desdiani:** conceptualization, investigation, formal analysis, writing – original draft. **Nurjati Chairani Siregar:** conceptualization, formal analysis, writing – review and editing. **Asysyukriati Prawiro:** conceptualization, formal analysis, writing – review and editing. **Khansa Kiasati Chandra Syahbunan:** conceptualization, formal analysis, writing – review and editing. All authors have accepted responsibility for the entire content of this manuscript and approved its submission.

## Funding

The authors have nothing to report.

## Consent

The authors declare that written informed consent was obtained for the publication of this manuscript and accompanying images using the consent forms provided by the Journal.

## Conflicts of Interest

The authors declare no conflicts of interest.

## Data Availability

The data that support the findings of this study are available on request from the corresponding author. The data are not publicly available due to privacy or ethical restrictions.
